# Post-infection cognitive impairments in a cohort of elderly patients with COVID-19

**DOI:** 10.1186/s13024-021-00469-w

**Published:** 2021-07-19

**Authors:** Yu-Hui Liu, Ye-Ran Wang, Qing-Hua Wang, Yang Chen, Xian Chen, Ying Li, Yuan Cen, Cheng Xu, Tian Hu, Xu-Dong Liu, Ling-Li Yang, Si-Jing Li, Xue-Fei Liu, Chun-Mei Liu, Jie Zhu, Wei Li, Li-Li Zhang, Juan Liu, Yan-Jiang Wang

**Affiliations:** 1 Department of Neurology and Centre for Clinical Neuroscience, Daping Hospital, Third Military Medical University, Chongqing, China; 2 Department of Anaesthesiology, Daping Hospital, Third Military Medical University, Chongqing, China; 3 Department of Ophthalmology, Daping Hospital, Third Military Medical University, Chongqing, China; 4 Department of Orthopedics, Daping Hospital, Third Military Medical University, Chongqing, China; 5 Department of Oncology, General Hospital of the Central Theatre Command of the People’s Liberation Army, Wuhan, China

**Keywords:** COVID-19, Coronavirus, Cognitive impairment, Post infection

## Abstract

**Background:**

Understanding the long-term effects of coronavirus disease 2019 (COVID-19) on cognitive function is essential for monitoring the cognitive decline in the elderly population. This study aims to assess the current cognitive status and the longitudinal cognitive decline in elderly patients recovered from COVID-19.

**Methods:**

This cross-sectional study recruited 1539 COVID-19 inpatients aged over 60 years who were discharged from three COVID-19-designated hospitals in Wuhan, China, from February 10 to April 10, 2020. In total, 466 uninfected spouses of COVID-19 patients were selected as controls. The current cognitive status was assessed using a Chinese version of the Telephone Interview of Cognitive Status-40 (TICS-40) and the longitudinal cognitive decline was assessed using an Informant Questionnaire on Cognitive Decline in the Elderly (IQCODE). Cognitive assessments were performed 6 months after patient discharge.

**Results:**

Compared with controls, COVID-19 patients had lower TICS-40 scores and higher IQCODE scores [TICS-40 median (IQR): 29 (25 to 32) vs. 30 (26 to 33), *p* < 0.001; IQCODE median (IQR): 3.19 (3.00 to 3.63) vs. 3.06 (3.00 to 3.38), *p* < 0.001]. Severe COVID-19 patients had lower TICS-40 scores and higher IQCODE scores than non-severe COVID-19 patients [TICS-40 median (IQR): 24 (18 to 28) vs. 30 (26 to 33), *p* < 0.001; IQCODE median (IQR): 3.63 (3.13 to 4.31) vs. 3.13 (3.00 to 3.56), *p* < 0.001] and controls [TICS-40 median (IQR): 24 (18 to 28) vs. 30 (26 to 33), *p* < 0.001; IQCODE median (IQR) 3.63 (3.13 to 4.31) vs. 3.06 (3.00 to 3.38), *p* < 0.001]. Severe COVID-19 patients had a higher proportion of cases with current cognitive impairment and longitudinal cognitive decline than non-severe COVID-19 patients [dementia: 25 (10.50 %) vs. 9 (0.69 %), *p* < 0.001; Mild cognitive impairment (MCI): 60 (25.21 %) vs. 63 (4.84 %), *p* < 0.001] and controls [dementia: 25 (10.50 %) vs. 0 (0 %), *p* < 0.001; MCI: 60 (25.21 %) vs. 20 (4.29 %), *p* < 0.001)]. COVID-19 severity, delirium and COPD were risk factors of current cognitive impairment. Low education level, severe COVID-19, delirium, hypertension and COPD were risk factors of longitudinal cognitive decline.

**Conclusions:**

Severe acute respiratory syndrome coronavirus 2 (SARS-CoV-2) infection is associated with an increased risk of long-term cognitive decline in elderly population. COVID-19 patients, especially severe patients, should be intensively monitored for post-infection cognitive decline.

**Supplementary Information:**

The online version contains supplementary material available at 10.1186/s13024-021-00469-w.

## Background

The coronavirus disease 2019 (COVID-19) pandemic has affected over 180 million patients thus far, and the number is still increasing [1]. Understanding the short- and long-term health consequences of COVID-19 is therefore critical. Severe acute respiratory syndrome coronavirus 2 (SARS-CoV-2) infection causes damages to multiple systems, including the respiratory, digestive, cardiovascular, renal, immune and nervous systems [2]. More than one-third of hospitalized patients with COVID-19 experience a variety of neurologic manifestations at the acute stage of the infection, including altered cognitive and mental status, cerebrovascular diseases, headache, vertigo, anosmia and ageusia [3], and neurological sequelae are also reported [4–7]. Acute cognitive complications are common [8]; however, long-term effects of COVID-19 on cognition are not clear yet. The first bulk of hospitalized COVID-19 patients in Wuhan, China, has recovered for half a year. This study aimed to investigate the long-term impact of SARS-CoV-2 infection on cognitive changes 6 months after recovery, and to determine risk factors of cognitive impairment in elderly patients recovered from COVID-19.

## Methods

### Participants

 Participants in this study were inpatients who were discharged between February 10 and April 10, 2020, from three hospitals, including Huoshenshan Hospital, Tongji Taikang Hospital and General Hospital of the Central Theatre Command of the People’s Liberation Army, which were designated to treat COVID-19 patients in Wuhan during the pandemic in early 2020. Eligibility included the following: (1) aged 60 years and older; (2) agreed to participate in this study. Subjects were excluded if they had the following conditions: (1) did not agree to participate, did not understand the questions in the questionnaires, or had communicative obstacles due to language or hearing reasons; (2) pre-existing subjective or diagnosed dementia; (3) a family history of dementia which may increase the risk of cognitive impairment; (4) a concomitant neurologic disorder potentially affecting cognitive function; and (5) severe cardiac, hepatic, renal diseases or any kind of tumour. Uninfected spouses that co-lived with the patients in the same environment were selected as controls. In total, 3233 patients discharged from the hospitals during the designated study period were screened.

Ultimately, 1539 patients, including 238 severe cases and 1301 non-severe cases, were eligible for this survey, and 466 spouses were recruited as controls. COVID-19 designated hospitals including Huoshenshan and Tongji Taikang Hospital were disbanded after the crisis. Therefore, the protocols were approved by the institutional review boards of Daping Hospital, Third Military Medical University, which launched this study. Since this study is conducted based on telephone interviews, written consents were waived but verbal informed consents were obtained from all participants or their legal guardians prior to the survey.

### Clinical and cognitive assessment

The diagnosis of COVID-19 was based on the World Health Organization interim guidance [9]. The severity of COVID-19 was defined as severe or non-severe following the American Thoracic Society guidelines for community-acquired pneumonia [10]. Accordingly, severe cases with COVID-19 were defined as: fever or suspected respiratory infection, plus at least one of the following conditions: respiratory rate > 30 breaths/min, severe respiratory distress, or SpO2 < 90 % on room air. Uninfected spouses were confirmed to be uninfected by high-throughput sequencing or real-time reverse-transcriptase polymerase-chain-reaction assay (RT-PCR) for nasal and pharyngeal swab specimens.

The survey was conducted 6 months after patient discharge. Due to the emerging infection risk, participants were interviewed by a telephone survey. The telephone interview was conducted by a group of trained raters. Participants were allowed to terminate the survey at any time.

The following information was collected from medical records and a knowledgeable family member for each participant: demographics, including age, sex, education level, body mass index (BMI), comorbidities, including hypertension, diabetes mellitus, coronary heart disease, hyperlipidaemia, a history of cardiovascular diseases or stroke, and chronic obstructive pulmonary disease (COPD). The current cognitive status of participants was assessed using a Chinese version of the Telephone Interview of Cognitive Status-40 (TICS-40, Supplementary Table 1), which was previously validated [11]. The TICS-40 includes 10 variables and has a maximum of 40 points. Score ≤ 20 was determined as mild cognitive impairment (MCI), and score ≤ 12 was determined as dementia [11]. Subjects’ family informants were interviewed to report the cognitive decline of patients and their spouses over the previous 6 months using the Chinese version of the short form Informant Questionnaire on Cognitive Decline in the Elderly (IQCODE, Supplementary Table 2) [12], which contains 16 items that rate changes in memory and other cognitive domains and was previously validated [13]. Cognitive decline was defined as an IQCODE score ≥ 3.5, which provided a sensitivity of 92 % and a specificity of 80 % [12].

### Quality control of cognitive assessment

A group of raters consisting of six experienced nurses were trained with the questionnaires by a neurologist experienced in cognitive assessments. Seven subjects with dementia, five subjects with MCI and nine cognitively normal subjects were recruited for the interrater reliability assessment. The intraclass correlation coefficient (ICC) was 0.990 for TICS and 0.934 for IQCODE, reflecting a high interrater reliability for the assessments.

### Statistical analysis

Continuous variables, including age, education, BMI, TICS-40 and IQCODE, were not normally distributed, thus Mann-Whitney U tests were used to compare these variables between groups, and Kruskal Wallis tests were used for multigroup comparison. Two-sample tests of proportions (for categorical data) were used to compare proportions. As for comparison of TICS-40 and IQCODE, variables including sex, age, education, BMI and comorbidities that were significantly different among groups were adjusted for potential confounding effects.

Linear and logistic regression models were utilized to assess the association between COVID-19 and cognitive outcomes. Mechanical ventilation was excluded for regression analyses to reduce collinearity with COVID-19 severity. In linear regression models, cognitive outcomes (TICS and IQCODE) were fitted as continuous variables. The linear regression models were adjusted for age and sex, and in the next step, variables including education level, BMI, COVID-19 severity, intensive care unit (ICU) admission, high flow oxygen therapy, delirium, hypertension, diabetes mellitus, hyperlipidaemia, stroke history, coronary heart disease, and COPD were added into the adjusted models. In logistical regression models, we first fitted univariate models with a single candidate variable at one time, with the cross-sectional cognitive status (TICS-40 ≤ 20 indicates cognitive impairment) or longitudinal cognitive change (IQCODE ≥ 3.5 indicates cognitive decline) as the dependent variable and education level, BMI, COVID-19 severity, ICU admission history, delirium, hypertension, diabetes mellitus, hyperlipidaemia, COPD, or history of stroke and coronary heart disease, as the independent variable. Potential risk factors with a *P* value less than 0.2 in univariate analyses were included in the final multivariate regression model with adjustment for age and sex. Statistical analyses were conducted using SPSS statistical package version 24 (IBM SPSS Statistics for Windows, Armonk, NY, USA).

## Results

### Demographics of subjects

**Fig. 1:**
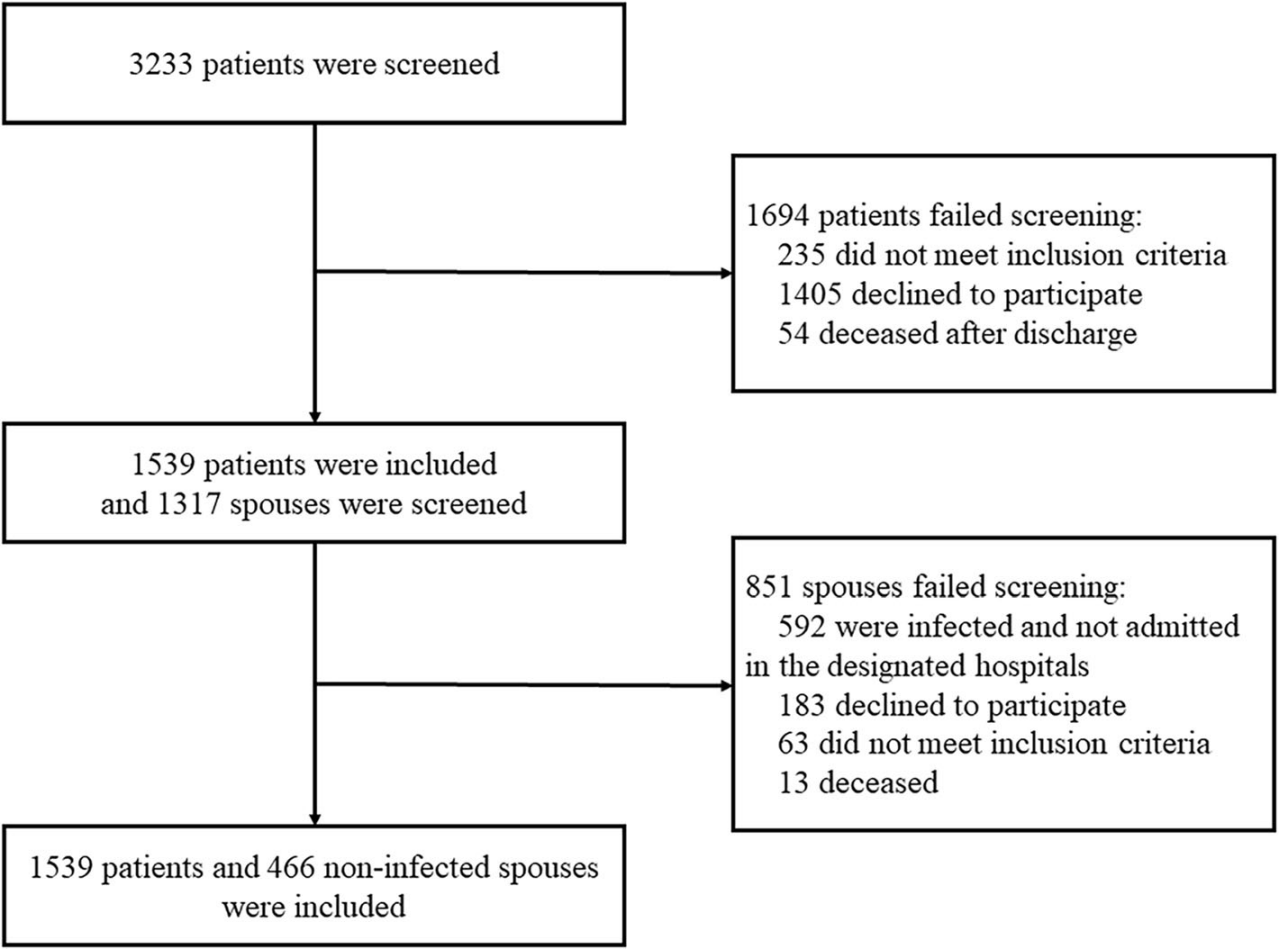
Participants screening flowchart. In total, 3233 subjects were screened for eligibility and finally 1539 patients were included in this study. 1365 spouses were screened and finally 466 non-infected spouses were selected as controls. The reasons for exclusion were specified in the flow chart

In total, 3233 COVID-19 patients aged 60 years or above were screened for eligibility for this study. 1694 patients failed screening, among which 235 patients did not meet the inclusion criteria, 1405 patients declined to participate and 54 patients died after discharge. Finally, 1539 patients were enrolled in this study. Among 1317 co-living spouses, 851 spouses failed screening due to the following reasons: 592 spouses were infected with SARS-CoV-2 and not admitted in the three designated hospitals, thus their demographic and medical information were not obtained. 183 spouses declined to participate, 63 spouses did not meet the inclusion criteria and 13 spouses died (Fig. 1). Finally, 466 spouses were included in this study.

There were no significant differences in the mean age, frequencies of males, median education level and BMI between COVID-19 patients and controls. Moreover, frequencies of hypertension, diabetes mellitus, hyperlipidaemia, COPD, stroke, and coronary heart disease in COVID-19 patients were comparable to those in controls. Compared with non-severe COVID-19 patients, severe COVID-19 patients were older, had a higher proportion of males and a higher BMI, and more frequently had hypertension, a history of stroke, coronary heart disease, and COPD. Severe COVID-19 patients had comparable frequencies of diabetes mellitus and hyperlipidaemia with controls. Compared with non-severe patients, severe cases had higher education levels. Moreover, severe cases had higher frequencies of ICU admission, receiving mechanical ventilation, high flow oxygen therapy and incidences of delirium during hospitalization than non-severe cases (Table 1). Furthermore, significant differences were not found in the demographic characteristics, including age, sex, education levels, BMI, incidences of severe COVID-19, frequencies of hypertension, diabetes mellitus, hyperlipidaemia, stroke history, coronary heart disease and COPD, frequencies of ICU admission, receiving mechanical ventilation, high flow oxygen therapy and incidences of delirium, between patients included and those not included in this study (Table 2).

**Table 1:** Demographic data of subjects

	Patients (*n* = 1539)	Severe cases (*n* = 238)	Non-severe cases (*n* = 1301)	Control (*n* = 466)	*P* value Patients vs. Control	*P* value Severe vs. Non-Severe
Age – Median (IQR), year	69 (66, 75)	72(67–80)	68(66–74)	67(66–76)	0.119^a^	< 0.001^a^
Male – No. (%)	738 (47.95)	128(53.8)	607(46.7)	226(48.5)	0.761^b^	0.041^b^
Education – Median (IQR), year	12 (9–12)	12(6–12)	12(9–12)	12(6–12)	0.758^a^	0.003 ^a^
BMI – Median (IQR), kg/m^2^	24.0(22.5–25.4)	24.4(22.9–25.7)	23.9(22.4–25.3)	24.2(22.5–25.6)	0.407^a^	0.002^a^
Coexisting disorders – No. (%)						
Hypertension	589 (38.27)	112(47.1)	477(36.7)	164(35.2)	0.229^b^	0.002^b^
Diabetes mellitus	304 (19.75)	52(21.8)	252(19.4)	87(18.7)	0.605^b^	0.377^b^
Hyperlipidemia	155 (10.07)	27(11.3)	128(9.8)	42(9.0)	0.501^b^	0.478^b^
Stroke history	87 (5.65)	40(16.8)	47(3.6)	36(7.7)	0.102^b^	< 0.001^b^
Coronary heart disease history	212 (13.78)	66(27.7)	146(11.2)	67(14.4)	0.704^b^	< 0.001^b^
COPD	150 (9.75)	42(17.6)	108(8.3)	42(9.0)	0.637^b^	< 0.001^b^
ICU admission – No. (%)	81 (5.26)	56(23.5)	24(1.8)			< 0.001^b^
Mechanical Ventilation, No. (%)	96 (6.24)	62(26.1)	33(2.5)			< 0.001^b^
High flow oxygen therapy, No. (%)	326 (21.18)	93(39.1)	232(17.8)			< 0.001^b^
Delirium, No. (%)	105 (6.82)	61(25.6)	43(3.3)			< 0.001^b^

Abbreviation: *IQR* Inter-Quartile Range, *BMI* Body Mass Index, *ICU* Intensive Care Unit, *COPD* Chronic Obstructive Pulmonary Disease

^a^Mann-Whitney U test

^b^Pearson χ^2^ test

**Table 2:** Demographic data of subjects included and not included in this study

	Patients included (*n* = 1539)	Patients not included (*n* = 1694)	*P* value
Age – Median (IQR), year	69 (66, 75)	70 (66, 75)	0.165^a^
Male – No. (%)	738 (47.95)	826 (48.76)	0.647^b^
Education – Median (IQR), year	12 (9–12)	12 (9–12)	0.226^a^
BMI – Median (IQR)	23.97 (22.52, 25.38)	23.92 (22.42, 25.41)	0.284^a^
Severe cases -No. (%)	238 (15.46)	257 (15.17)	0.845^b^
Coexisting disorders – No. (%)			
Hypertension	589 (38.27)	640 (37.78)	0.800^b^
Diabetes mellitus	304 (19.75)	348 (20.54)	0.599^b^
Hyperlipidaemia	155 (10.07)	176 (10.39)	0.772^b^
Stroke history	87 (5.65)	91 (5.37)	0.758^b^
Coronary heart disease history	212 (13.78)	231 (13.64)	0.919^b^
COPD	150 (9.75)	163 (9.62)	0.905^b^
ICU admission – No. (%)	81 (5.26)	94 (5.55)	0.756^b^
Mechanical Ventilation, No. (%)	96 (6.24)	106 (6.26)	1.000^b^
High flow oxygen therapy, No. (%)	326 (21.18)	355 (20.96)	0.897^b^
Delirium, No. (%)	105 (6.82)	117 (6.91)	0.945^b^

Abbreviation: *IQR* Inter-Quartile Range, *BMI* Body Mass Index, *ICU* intensive care unit, *COPD* Chronic Obstructive Pulmonary Disease

^a^Mann-Whitney U test

^b^Pearson χ^2^ test

### Current cognitive impairment of COVID-19 patients

**Fig. 2:**
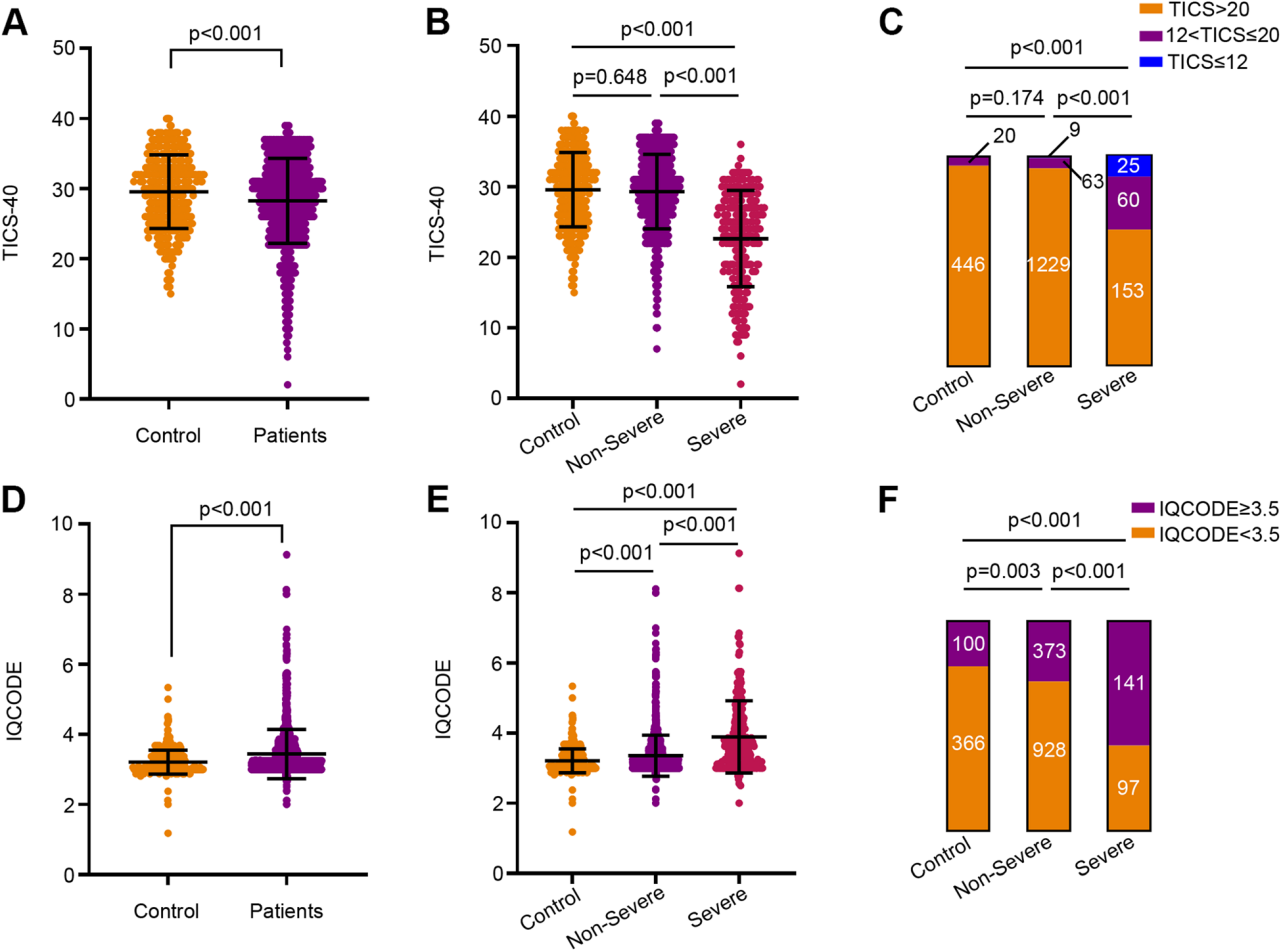
Current cognitive status and longitudinal cognitive decline in COVID-19 patients and controls. **A** TICS-40 scores of COVID-19 patients and controls. **B** TICS-40 scores of severe and non-severe cases with COVID-19 and controls. **C** Composition of subjects with different TICS-40 scores in severe and non-severe cases with COVID-19 and controls. **D** IQCODE scores of COVID-19 patients and controls. **E** IQCODE scores of severe and non-severe cases with COVID-19 and controls. **F** Composition of subjects with different IQCODE scores in severe and non-severe cases with COVID-19 and controls. **A** and **D**, Wilcoxon-Mann Whitney test. **B** and **E**, Kruskal Wallis test. **C**, Fisher exact test. **F**, χ^2^ test

TICS-40 was utilized to determine the current cognitive status of participants. COVID-19 patients had lower TICS-40 scores than controls [median (IQR): 29 (25 to 32) vs. 30 (26 to 33), *p* < 0.001] (Fig. 2A). Severe COVID-19 patients had lower TICS-40 scores than non-severe patients [median (IQR): 24 (18 to 28) vs. 30 (26 to 33), *p* < 0.001] and controls [median (IQR): 24 (18 to 28) vs. 30 (26 to 33), *p* < 0.001]. TICS-40 scores were comparable between non-severe COVID-19 cases and controls (*p* = 0.648) (Fig. 2B). Furthermore, the differences remained significant after adjusted for potential confounding factors (Supplementary Tables 3 and 4).

A cut-off value of 20 was defined for MCI and 12 for dementia [13]. Severe COVID-19 patients more frequently reported to have dementia and MCI than non-severe COVID-19 patients [dementia: 25 (10.50 %) vs. 9 (0.69 %), *p* < 0.001; MCI: 60 (25.21 %) vs. 63 (4.84 %), *p* < 0.001] and controls [dementia: 25 (10.50 %) vs. 0 (0 %), *p* < 0.001; MCI: 60 (25.21 %) vs. 20 (4.29 %), *p* < 0.001)]. However, no difference was found in the proportion of cases with dementia or MCI between non-severe COVID-19 patients and controls (dementia: *p* = 0.703; MCI: *p* = 0.123) (Fig. 2C). These findings indicate that current cognitive impairment was associated with both SARS-CoV-2 infection and severity of COVID-19. Furthermore, differences were still significant after adjusted for potential confounding factors.

### Longitudinal cognitive decline of COVID-19 patients

At 6 months after discharge, the cognitive decline of COVID-19 patients and their non-infected spouses was assessed with the IQCODE, with higher IQCODE scores indicating larger cognitive decline. COVID-19 patients had higher IQCODE scores than controls [median (IQR): 3.19 (3.00 to 3.63) vs. 3.06 (3.00 to 3.38), *p* < 0.001] (Fig. 2D). Moreover, severe COVID-19 cases had higher IQCODE scores than non-severe cases [median (IQR): 3.63 (3.13 to 4.31) vs. 3.13 (3.00 to 3.56), *p* < 0.001] and controls [median (IQR): 3.63 (3.13 to 4.31) vs. 3.06 (3.00 to 3.38), *p* < 0.001]. Non-severe cases had higher IQCODE scores than controls (*p* < 0.001) (Fig. 2E). Furthermore, differences remained significant after adjusted for potential confounding factors (Supplementary Tables 5 and 6).

Based on a cut-off value of 3.5 for clinically meaningful cognitive decline [12], severe COVID-19 patients more frequently showed cognitive decline than non-severe COVID-19 patients [141 (59.24 %) vs. 373 (28.67 %), *p* < 0.001] and controls [141 (59.24 %) vs. 100 (21.46 %), *p* < 0.001]. Meanwhile, non-severe COVID-19 patients also more frequently showed cognitive decline than controls [373 (28.67 %) vs. 100 (21.46 %), *p* = 0.003] (Fig. 2F). These findings indicate that both SARS-CoV-2 infection and severity of COVID-19 are associated with longitudinal cognitive decline. Furthermore, differences were still significant after adjusted for potential confounding factors.

### Risk factors for cognitive impairment and decline in COVID-19 patients

Regression models were used to investigate potential risk factors of current cognitive impairment or post-infection cognitive decline in COVID-19 patients. In univariate logistical regression analyses, age, severe COVID-19, ICU admission, delirium, stroke history, coronary heart disease and COPD were associated with cognitive impairment. Severe of COVID-19, delirium and COPD remained associated with current cognitive impairment in the multivariate model (Table 3). In the linear regression model with adjustment for age and sex, COVID-19 severity, ICU admission, delirium, and COPD were associated with lower TICS-40 scores. Higher education level and high flow oxygen therapy were associated with higher TICS-40 scores (Supplementary Table 7).

**Table 3:** Logistic regression models to evaluate risk factors for cognitive impairment as indicated by TICS-40 ≤ 20 in COVID-19 patients

Variables	Univariable ORs(95 %CI)	*P* value	Multivariable ORs(95 %CI)	*P* value
Age, year	1.030(1.009–1.052)	0.005		
Sex, male	1.235(0.887–1.721)	0.212		
Education, year	1.000(0.971–1.030)	0.987		
BMI, kg/m^2^	1.055(0.957–1.163)	0.279		
Severe, vs. non-severe	9.311(6.518–13.300)	< 0.001	6.507(4.411–9.599)	< 0.001
ICU admission, vs. no	8.601(5.326–13.890)	< 0.001		
High flow oxygen therapy, vs. no	1.901(1.324–2.727)	< 0.001		
Delirium, vs. no	7.850(5.076–12.139)	< 0.001	3.714(2.247–6.136)	< 0.001
Coexisting disorder, vs. no				
Hypertension	1.278(0.915–1.787)	0.150		
Diabetes	1.149(0.768–1.718)	0.500		
Hyperlipidaemia	1.023(0.592–1.766)	0.935		
Stroke history	3.329(1.989–5.572)	< 0.001		
Coronary heart disease	1.725(1.134–2.622)	0.011		
COPD	4.733(3.169–7.068)	< 0.001	4.224(2.693–6.625)	< 0.001

Dependent variables: TICS-40 score ≤ 20

Independent variables: age, sex, severity, ICU admission, high flow oxygen therapy, delirium, hypertension, stroke, coronary heart disease, COPD

Abbreviations: *BMI* body mass index, *ICU* intensive care unit, *COPD* Chronic Obstructive Pulmonary Disease

Consistently, in univariate logistical regression analyses, age, lower education level, severe COVID-19, ICU admission, delirium, hypertension, diabetes, stroke history, coronary heart disease and COPD were associated with longitudinal cognitive decline. In the multivariate model with adjustment for age and sex, the association remained significant for lower education level, severe COVID-19, delirium, hypertension and COPD. However, high flow oxygen therapy was protective against longitudinal cognitive decline (Table 4). In the linear regression mode adjusted for age and sex, COVID-19 severity, ICU admission, delirium, hypertension, hyperlipidaemia and COPD were found to be associated with higher IQCODE scores. However, high flow oxygen therapy was found to be associated with lower IQCODE scores (Supplementary Table 8).

**Table 4:** Logistic regression models to evaluate the risk factors for longitudinal cognitive decline as indicated by IQCODE score ≥ 3.5 in COVID-19 patients

Variables	Univariable ORs (95 %CI)	*P* value	Multivariable ORs (95 %CI)	*P* value
Age, year	1.023(1.009–1.037)	0.001		
Sex, male	0.934(0.756–1.157)	0.530		
Education, year	0.959(0.936–0.982)	0.001	0.968(0.944–0.993)	0.011
BMI, kg/m^2^	1.007(0.947–1.072)	0.821		
Severe, vs. non-severe	3.616(2.719–4.810)	< 0.001	2.833(2.065–3.888)	< 0.001
ICU admission, vs. no	5.100(3.122–8.331)	< 0.001		
High flow oxygen therapy, vs. no	0.828(0.635–1.079)	0.163	0.421(0.302–0.586)	< 0.001
Delirium, vs. no	5.578(3.597–8.650)	< 0.001	5.480(3.292–9.122)	< 0.001
Coexisting disorders, vs. no				
Hypertension	1.825(1.470–2.266)	< 0.001	1.661(1.320–2.091)	< 0.001
Diabetes	1.344(1.037–1.743)	0.026		
Hyperlipidaemia history	1.180(0.835–1.666)	0.348		
Stroke history	1.590(1.027–2.463)	0.038		
Coronary heart diseases	1.800(1.341–2.415)	< 0.001		
COPD	2.368(1.686–3.326)	< 0.001	2.005(1.398–2.877)	< 0.001

Dependent variable: IQCODE ≥ 3.5

Independent variables: age, sex, education, severity, ICU admission, high flow oxygen therapy, delirium, hypertension, diabetes, stroke, coronary heart disease, COPD

Abbreviations: *BMI* body mass index, *ICU *intensive care unit, *COPD* Chronic Obstructive Pulmonary Disease

## Discussion

Currently, the association between COVID-19 and long-term cognitive change post-infection has rarely been investigated. We found that COVID-19 patients, including both severe and non-severe cases, had worse cognitive outcomes 6 months after recovery, indicating that SARS-CoV-2 infection may affect long-term cognitive performance, particularly in severe patients, among which 35.71 % of patients had current cognitive impairment, and 59.24 % reported longitudinal cognitive decline. This study identified several risk factors for post-infection cognitive impairment in COVID-19 patients, including older age, lower education level, comorbidities, severe COVID-19, ICU admission and delirium.

While the exact mechanism underlying this association remains to be elucidated, the aetiology of cognitive decline post-SARS-CoV-2 infection may be multifactorial. First, a potent mechanism underlying cognitive decline after SARS-CoV-2 infection is hypoxia [2], because brain regions associated with cognitive functions, such as the hippocampus, are susceptible to hypoxia induced neuronal damage [14, 15]. Oxygen deficiency at the acute disease stage and after recovery can cause damages to neurons, which are sensitive to hypoxia [16]. This hypothesis is evidenced by our finding that severe COVID-19 patients had worse cognitive impairment, as these patients were in a more hypoxic state even months after recovery [1, 17]. Moreover, the severity of COVID-19 and ICU admission, were found to be associated with an increased risk of cognitive impairment. A previous study demonstrated that cognitive sequelae occurred in patients who survived acute respiratory distress syndrome (ARDS), with a rate of 73 % at hospital discharge, 46 % at 1 year, and 47 % at 2 years after discharge, indicating that severe COVID-19 disease, which is commonly complicated by ARDS, might affect long-term cognitive performance [18]. This is also supported by our finding that high flow oxygen therapy during acute phase of COVID-19, which may alleviate oxygen deficiency, could be protective against post-infection cognitive decline. Moreover, COVID-19 might also prompt neuronal injury through vascular impairment, which could lead to ischaemia and damage cognitive function. This rationale is supported by a recent study which found that COVID-19 patients had an increased risk of stroke [19]. Second, acute and chronic systemic inflammation and immune dysregulation after SARS-CoV-2 infection might also cause damage to the brain and thus may lead to cognitive decline [19]. It is speculated that the inflammatory status after SARS-CoV-2 infection may promote neuronal damage and accelerate the pathogenesis of neurodegenerative diseases [17]. This mechanism might explain why older age was associated with an increased risk of cognitive impairment in our cohort. Third, it is possible that SARS-CoV-2 can directly infect the brain via the blood-brain barrier, olfactory nerve and infiltration of infected immune cells [19, 20]. Brain infection could lead to encephalopathy and encephalitis [21–23]. In addition, invasion of SARS-CoV-2 might prompt cytotoxic aggregation of proteins, including amyloid-β and α-synuclein [24], which may promote post-infection neurodegeneration. This hypothesis is reinforced by a recent finding that SARS-CoV-2 infection induces hypometabolism in brain areas that are generally affected by neurodegenerative diseases [25]. Increased production of amyloid-β and α-synuclein might be attributed to a physical reaction for their anti-infection capacity [26, 27]. In this study, high education level was found to be a protective factor against cognitive decline in COVID-19 patients, which is consistent with previous studies [28, 29]. It is suggested that more education did not protect individuals from developing neurodegenerative neuropathology but it appears to mitigate the impact of pathology on the clinical expression of dementia, which is coined as “functional protection” [30, 31].

There were several limitations in the present study. First, telephone interviews are not accurate as traditional face-to-face cognitive assessments. This is an alternative choice due to the possible emerging infection risk. Second, pre-infection cognitive information of COVID-19 patients were not available, thus comparison of pre- and post-infection cognitive status with objective assessments cannot be done. This might be an inherent deficit of this kind of studies. Instead, the IQCODE was used to evaluate the longitudinal cognitive decline of these patients. IQCODE is a widely used tool to assess the longitudinal cognitive decline under circumstances where the baseline information is lacking [32]. However, even the use of IQCODE could not completely avoid the biases as relatives may tend to report a cognitive decline in severe COVID-19 patients. Third, we did not include a group of patients with non-COVID-19 pneumonia at the same period, as these patients with pneumonia were less likely admitted to hospitals during this pandemic. Therefore, the specificity of COVID-19 associated cognitive decline could not be addressed. We could not determine disease types of the cognitive decline in our cohort at the current stage, this will be addressed in future longitudinal studies. Furthermore, it is possible that patients declined to participate in this study as they do not experience cognitive complaints, which could be a potential selection bias. In this study, co-living, non-infected spouses of COVID-19 patients were selected as the controls. As their ages, sex composition, education levels, living conditions and lifestyles were similar to those of patients, this control selection could help to reduce the bias attributed to these factors which may affect cognitive function. It is also possible that the un-infected spouses who were resistant to COVID-19 infection were healthier and less likely to be demented. Moreover, sample sizes between the patients and controls do not match, which may influence the findings of this study.

## Conclusions

This cross-sectional study suggests that SARS-CoV-2 infection has a potential long-term impact on the cognition of patients. As the COVID-19 pandemic is still raging in many countries and is expected to last for a long period, the long-term cognitive sequelae may become a major public health issue long after the pandemic has ended. Longitudinal studies to follow up patients who have recovered from COVID-19 are necessary for better understanding the long-term cognitive consequences of COVID-19, particularly among those who have recovered from severe disease.

## Supplementary Information

**Additional file 1: Supplemental Table 1.** Telephone Interview of Cognitive Status-40 (TICS-40). **Supplemental Table 2.** Short Form of the Informant Questionnaire on Cognitive Decline in the Elderly (IQCODE). **Supplemental Table 3. **A linear regression model to adjust for confounding factors in Fig. 2A. **Supplemental Table 4. **Linear regression models to adjust for confounding factors in Fig. 2B. **Supplemental Table 5.** A linear regression model to adjust for confounding factors in Fig. 2D. **Supplemental Table 6. **Linear regression models to adjust for confounding factors in Fig. 2E. **Supplemental Table 7.** A linear regression model to evaluate risk factors for cognitive impairment as indicated by TICS-40. **Supplemental Table 8. **A linear regression model to evaluate risk factors for cognitive decline as indicated by IQCODE.

## Data Availability

The authors are open to sharing statistical codes and study data.

## Abbreviations

COVID-19: Coronavirus disease 2019
SARS-CoV-2: Severe acute respiratory syndrome coronavirus 2
RT-PCR: Reverse-transcriptase polymerase-chain-reaction assay
BMI: Body mass index
COPD: Chronic obstructive pulmonary disease
TICS-40: Telephone Interview of Cognitive Status-40
IQCODE: Informant Questionnaire on Cognitive Decline in the Elderly
MCI: Mild cognitive impairment
ICC: Intraclass correlation coefficient
ICU: Intensive care unit
ARDS: Acute respiratory distress syndrome
