# Supplementary material for: Post-infection cognitive impairments in a cohort of elderly patients with COVID-19
Source: Mol Neurodegener. 2021 Jul 19;16:48. doi: 10.1186/s13024-021-00469-w (PMC8287105; doi:10.1186/s13024-021-00469-w)
Supplement: Supplementary file 1 — Additional file 1: Supplemental Table 1. Telephone Interview of Cognitive Status-40 (TICS-40). Supplemental Table 2. Short Form of the Informant Questionnaire on Cognitive Decline in the Elderly (IQCODE). Supplemental Table 3. A linear regression model to adjust for confounding factors in Fig. 2A. Supplemental Table 4. Linear regression models to adjust for confounding factors in Fig. 2B. Supplemental Table 5. A linear regression model to adjust for confounding factors in Fig. 2D. Supplemental Table 6. Linear regression models to adjust for confounding factors in Fig. 2E. Supplemental Table 7. A linear regression model to evaluate risk factors for cognitive impairment as indicated by TICS-40. Supplemental Table 8. A linear regression model to evaluate risk factors for cognitive decline as indicated by IQCODE. [file 13024_2021_469_MOESM1_ESM.docx]

**Supplementary information**

**Supplemental Table 1. Telephone Interview of Cognitive Status-40 (TICS-40).**

| **Orientation** | | Score |
| --- | --- | --- |
| 1. (i) What day of the week is it? | |  |
| (ii) What is today’s date? | Year (1 point) |  |
|  | Month (1 point) |  |
|  | Date (1 point) |  |
| (iii) What season are we in? | Season (1 point) |  |
| 2. What is your house number, street, and city? | House number (1 point) |  |
|  | Street (1 point) |  |
|  | City (1 point) |  |
| **Registration/Free Recall** | |  |
| 3. I’m going to read you a list of 10 words (as listed below). Please listen carefully and try to remember them. When I am done, tell me as many as you can in any order. Ready? Now, tell me all the words you can remember. | |  |
| Cabin (1 point) | |  |
| Pipe (1 point) | |  |
| Elephant (1 point) | |  |
| Chest (1 point) | |  |
| Silk (1 point) | |  |
| Theatre (1 point) | |  |
| Watch (1 point) | |  |
| Whip (1 point) | |  |
| Pillow (1 point) | |  |
| Giant (1 point) | |  |
| **Attention/Calculation** | |  |
| 4. Please take 7 away from 100 (1 point) | 93 |  |
| Now continue to take 7 away from what you have left over until I ask you to stop (1 point for each step) | 86 |  |
|  | 79 |  |
|  | 72 |  |
|  | 65 |  |
| 5. Please count backwards from 20 to 1 (2 points if completely correct on fist trial, 1 point if correct on second trial) | |  |
| **Comprehension, Semantic and Recent Memory** | |  |
| 6. What do people usually use to cut paper? | Scissors (1 point) |  |
| 7. What is the prickly green plant found in the desert? | Cactus (1 point) |  |
| 8. Who is the president of China now? | Correct Name (1 point) |  |
| 9. Who is the Prime Minister now? | Correct Name (1 point) |  |
| **Language/Repetition** | |  |
| 10. Please say this ‘no ifs, ands or buts” (1 point) | |  |
| **Delayed recall** | |  |
| Please repeat the list of 10 words I read earlier | |  |
| Cabin (1 point) | |  |
| Pipe (1 point) | |  |
| Elephant (1 point) | |  |
| Chest (1 point) | |  |
| Silk (1 point) | |  |
| Theatre (1 point) | |  |
| Watch (1 point) | |  |
| Whip (1 point) | |  |
| Pillow (1 point) | |  |
| Giant (1 point) | |  |
| **Total Score** | |  |

**Reference:** Fong TG, Fearing MA, Jones RN, et al. Telephone interview for cognitive status: Creating a crosswalk with the Mini-Mental State Examination. Alzheimers Dement. 2009;5(6):492-497.

**Supplemental Table 2. Short Form of the Informant Questionnaire on Cognitive Decline in the Elderly (IQCODE)**

| Compare with pre-infection state | 1 point | 2 points | 3 points | 4 points | 5 points | Score |
| --- | --- | --- | --- | --- | --- | --- |
| 1. Remembering things about family and friends e.g. occupations, birthdays, addresses | Much improved | A bit improved | Not much change | A bit worse | Much worse |  |
| 2. Remembering things that have happened recently | Much improved | A bit improved | Not much change | A bit worse | Much worse |  |
| 3. Recalling conversations a few days later | Much improved | A bit improved | Not much change | A bit worse | Much worse |  |
| 4. Remembering his/her address and telephone number | Much improved | A bit improved | Not much change | A bit worse | Much worse |  |
| 5. Remembering what day and month it is | Much improved | A bit improved | Not much change | A bit worse | Much worse |  |
| 6. Remembering where things are usually kept | Much improved | A bit improved | Not much change | A bit worse | Much worse |  |
| 7. Remembering where to find things which have been put in a different place from usual | Much improved | A bit improved | Not much change | A bit worse | Much worse |  |
| 8. Knowing how to work familiar machines around the house | Much improved | A bit improved | Not much change | A bit worse | Much worse |  |
| 9. Learning to use a new gadget or machine around the house | Much improved | A bit improved | Not much change | A bit worse | Much worse |  |
| 10. Learning new things in general | Much improved | A bit improved | Not much change | A bit worse | Much worse |  |
| 11. Following a story in a book or on TV | Much improved | A bit improved | Not much change | A bit worse | Much worse |  |
| 12. Making decisions on everyday matters | Much improved | A bit improved | Not much change | A bit worse | Much worse |  |
| 13. Handling money for shopping | Much improved | A bit improved | Not much change | A bit worse | Much worse |  |
| 14. Handling financial matters e.g. the pension, dealing with the bank | Much improved | A bit improved | Not much change | A bit worse | Much worse |  |
| 15. Handling other everyday arithmetic problems e.g. knowing how much food to buy, knowing how long between visits from family or friends | Much improved | A bit improved | Not much change | A bit worse | Much worse |  |
| 16. Using his/her intelligence to understand what's going on and to reason things through | Much improved | A bit improved | Not much change | A bit worse | Much worse |  |
| **Average Score** | | | | | |  |

**Reference:** Fuh JL, Teng EL, Lin KN, et al. The Informant Questionnaire on Cognitive Decline in the Elderly (IQCODE) as a screening tool for dementia for a predominantly illiterate Chinese population. Neurology. 1995;45(1):92-96.

**Supplemental Table 3. A linear regression model to adjust for confounding factors in Figure 2A.**

| **Variables** | **Βeta** | **SE** | **Standardized Βeta** | **P value** |
| --- | --- | --- | --- | --- |
| Intercept | 31.916 | 2.211 |  | <0.001 |
| Sex | 0.042 | 0.258 | 0.004 | 0.871 |
| Age | -0.040 | 0.017 | -0.056 | 0.017 |
| Education | 0.086 | 0.026 | 0.078 | 0.001 |
| BMI | 0.019 | 0.074 | 0.006 | 0.794 |
| Hypertension | -0.523 | 0.274 | -0.043 | 0.057 |
| Diabetes mellitus | -0.287 | 0.339 | -0.019 | 0.397 |
| Hyperlipidemia | 0.132 | 0.442 | 0.007 | 0.765 |
| Stroke | -2.260 | 0.547 | -0.092 | 0.000 |
| Coronary artery disease | -1.288 | 0.387 | -0.075 | 0.001 |
| COPD | -3.604 | 0.432 | -0.179 | <0.001 |
| **Group** | **-1.316** | **0.301** | **-0.094** | **<0.001** |

Demographic variables including sex, age, education, BMI, hypertension, diabetes mellitus, hyperlipidemia, stroke, coronary artery disease, COPD were included in this regression model to adjust potential factors which may confound the comparison of TICS-40 scores between patients and control groups. Abbreviations: BMI, body mass index; COPD, Chronic Obstructive Pulmonary Disease. Abbreviations: BMI, body mass index; COPD: chronic obstructive pulmonary disease.

**Supplemental Table 4. Linear regression models to adjust for confounding factors in Figure 2B.**

| **Variables** | **Βeta** | **SE** | **Standardized Βeta** | **P value** |
| --- | --- | --- | --- | --- |
| Intercept | 28.279 | 2.110 |  | <0.001 |
| Sex | 0.211 | 0.245 | 0.018 | 0.390 |
| Age | -0.015 | 0.016 | -0.022 | 0.332 |
| Education | 0.080 | 0.025 | 0.073 | 0.001 |
| BMI | 0.087 | 0.070 | 0.025 | 0.212 |
| Hypertension | -0.359 | 0.260 | -0.029 | 0.169 |
| Diabetes mellitus | -0.434 | 0.322 | -0.029 | 0.177 |
| Hyperlipidemia | 0.014 | 0.419 | 0.001 | 0.973 |
| Stroke | -1.159 | 0.524 | -0.047 | 0.027 |
| Coronary artery disease | -0.656 | 0.369 | -0.038 | 0.076 |
| COPD | -2.999 | 0.412 | -0.149 | 0.000 |
| **Group：non-severe vs. control** | **-0.377** | **0.292** | **-0.030** | **0.197** |
| **Group：severe vs. control** | **-6.318** | **0.438** | **-0.346** | **<0.001** |
| **Variables** | **Βeta** | **SE** | **Standardized Βeta** | **P value** |
| Intercept | 27.902 | 2.095 |  | <0.001 |
| Sex | 0.211 | 0.245 | 0.018 | 0.390 |
| Age | -0.015 | 0.016 | -0.022 | 0.332 |
| Education | 0.080 | 0.025 | 0.073 | 0.001 |
| BMI | 0.087 | 0.070 | 0.025 | 0.212 |
| Hypertension | -0.359 | 0.260 | -0.029 | 0.169 |
| Diabetes mellitus | -0.434 | 0.322 | -0.029 | 0.177 |
| Hyperlipidemia | 0.014 | 0.419 | 0.001 | 0.973 |
| Stroke | -1.159 | 0.524 | -0.047 | 0.027 |
| Coronary artery disease | -0.656 | 0.369 | -0.038 | 0.076 |
| COPD | -2.999 | 0.412 | -0.149 | <0.001 |
| **Group：severe vs. non-severe** | **-5.941** | **0.395** | **-0.325** | **<0.001** |
| **Group：control vs. non-severe** | **0.377** | **0.292** | **0.027** | **0.197** |

Demographic variables including sex, age, education, BMI, hypertension, diabetes mellitus, hyperlipidemia, stroke, coronary artery disease, COPD were included in this regression model to adjust potential factors which may confound the comparison of TICS-40 scores among control, non-severe and severe groups. Abbreviations: BMI, body mass index; COPD, Chronic Obstructive Pulmonary Disease. Abbreviations: BMI, body mass index; COPD: chronic obstructive pulmonary disease.

**Supplemental Table 5. A linear regression model to adjust for confounding factors in Figure 2D.**

| **Variables** | **Βeta** | **SE** | **Standardized Βeta** | **P value** |
| --- | --- | --- | --- | --- |
| Intercept | 2.971 | 0.243 |  | <0.001 |
| Sex | 0.019 | 0.028 | 0.015 | 0.506 |
| Age | 0.005 | 0.002 | 0.061 | 0.011 |
| Education | -0.003 | 0.003 | -0.023 | 0.324 |
| BMI | -0.007 | 0.008 | -0.019 | 0.369 |
| Hypertension | 0.131 | 0.030 | 0.099 | <0.001 |
| Diabetes mellitus | 0.043 | 0.037 | 0.026 | 0.250 |
| Hyperlipidemia | 0.141 | 0.049 | 0.065 | 0.004 |
| Stroke | 0.101 | 0.060 | 0.038 | 0.094 |
| Coronary artery disease | 0.076 | 0.043 | 0.041 | 0.074 |
| COPD | 0.229 | 0.048 | 0.105 | <0.001 |
| **Group** | **0.224** | **0.033** | **0.147** | **<0.001** |

Demographic variables including sex, age, education, BMI, hypertension, diabetes mellitus, hyperlipidemia, stroke, coronary artery disease, COPD were included in this regression model to adjust potential factors which may confound the comparison of IQCODE scores between patients and control groups. Abbreviations: BMI, body mass index; COPD, Chronic Obstructive Pulmonary Disease. Abbreviations: BMI, body mass index; COPD: chronic obstructive pulmonary disease.

**Supplemental Table 6. Linear regression models to adjust for confounding factors in Figure 2E.**

| **Variables** | **Βeta** | **SE** | **Standardized Βeta** | **P value** |
| --- | --- | --- | --- | --- |
| Intercept | 3.268 | 0.238 |  | <0.001 |
| Sex | 0.005 | 0.028 | 0.004 | .864 |
| Age | 0.003 | 0.002 | 0.035 | .132 |
| Education | -0.002 | 0.003 | -0.019 | .402 |
| BMI | -0.013 | 0.008 | -0.034 | .103 |
| Hypertension | 0.118 | 0.029 | 0.088 | .000 |
| Diabetes mellitus | 0.055 | 0.036 | 0.034 | .130 |
| Hyperlipidemia | 0.150 | 0.047 | 0.069 | .001 |
| Stroke | 0.011 | 0.059 | 0.004 | .852 |
| Coronary artery disease | 0.024 | 0.042 | 0.013 | .558 |
| COPD | 0.180 | 0.046 | 0.082 | <0.001 |
| **Group：non-severe vs. control** | **0.147** | **0.033** | **0.109** | **<0.001** |
| **Group：severe vs. control** | **0.633** | **0.049** | **0.317** | **<0.001** |
| **Variables** | **Βeta** | **SE** | **Standardized Βeta** | **P value** |
| Intercept | 3.416 | 0.236 |  | <0.001 |
| Sex | 0.005 | 0.028 | 0.004 | 0.864 |
| Age | 0.003 | 0.002 | 0.035 | 0.132 |
| Education | -0.002 | 0.003 | -0.019 | 0.402 |
| BMI | -0.013 | 0.008 | -0.034 | 0.103 |
| Hypertension | 0.118 | 0.029 | 0.088 | 0.000 |
| Diabetes mellitus | 0.055 | 0.036 | 0.034 | 0.130 |
| Hyperlipidemia | 0.150 | 0.047 | 0.069 | 0.001 |
| Stroke | 0.011 | 0.059 | 0.004 | 0.852 |
| Coronary artery disease | 0.024 | 0.042 | 0.013 | 0.558 |
| COPD | 0.180 | 0.046 | 0.082 | <0.001 |
| **Group：non-severe vs. control** | **0.485** | **0.045** | **0.244** | **<0.001** |
| **Group：severe vs. control** | **-0.147** | **0.033** | **-0.097** | **<0.001** |

Demographic variables including sex, age, education, BMI, hypertension, diabetes mellitus, hyperlipidemia, stroke, coronary artery disease, COPD were included in this regression model to adjust potential factors which may confound the comparison of TICS-40 scores among control, non-severe and severe groups. Abbreviations: BMI, body mass index; COPD, Chronic Obstructive Pulmonary Disease. Abbreviations: BMI, Body mass index; COPD: Chronic obstructive pulmonary disease.

**Supplemental Table 7. A linear regression model to evaluate risk factors for cognitive impairment as indicated by TICS-40.**

| **Variables** | **Β** | **SE** | **Standardized Β** | **P value** |
| --- | --- | --- | --- | --- |
| Intercept | 33.521 | 1.466 |  | <0.001 |
| Sex | 0.347 | 0.277 | 0.029 | 0.210 |
| Age | 0.002 | 0.018 | 0.003 | 0.893 |
| Education | 0.094 | 0.027 | 0.087 | 0.001 |
| Severity | -5.469 | 0.412 | -0.326 | <0.001 |
| ICU admission | -2.394 | 0.861 | -0.088 | 0.005 |
| High flow oxygen therapy | 1.931 | 0.358 | 0.130 | <0.001 |
| Delirium | -2.805 | 0.751 | -0.116 | <0.001 |
| COPD | -3.772 | 0.460 | -0.185 | <0.001 |

This model is adjusted for age and sex. Abbreviations: ICU, Intensive care unit; COPD, Chronic obstructive pulmonary disease.

**Supplemental Table 8. A linear regression model to evaluate risk factors for cognitive decline as indicated by IQCODE.**

| **Variables** | **Β** | **SE** | **Standardized Β** | **P value** |
| --- | --- | --- | --- | --- |
| Intercept | 2.799 | 0.140 |  | <0.001 |
| Sex | 0.010 | 0.032 | 0.007 | 0.768 |
| Age | 0.003 | 0.002 | 0.034 | 0.139 |
| Severity | 0.276 | 0.049 | 0.142 | <0.001 |
| ICU admission | 0.252 | 0.102 | 0.080 | 0.013 |
| High flow oxygen therapy | -0.139 | 0.042 | -0.081 | 0.001 |
| Delirium | 0.835 | 0.089 | 0.298 | <0.001 |
| Hypertension | 0.113 | 0.033 | 0.078 | 0.001 |
| Hyperlipidaemia | 0.155 | 0.054 | 0.066 | 0.004 |
| COPD | 0.206 | 0.054 | 0.087 | <0.001 |

This model is adjusted for age and sex. Abbreviations: ICU, Intensive care unit; COPD, Chronic obstructive pulmonary disease.
